# Commentary: Identifying key predictors of post-stroke depression and cognitive impairment in acute stroke survivors

**DOI:** 10.3389/fneur.2026.1931929

**Published:** 2026-09-14

**Authors:** Polona Rus Prelog, Matija Zupan, Senta Frol

**Affiliations:** 1 Centre for Clinical Psychiatry, University Psychiatric Clinic, Ljubljana, Slovenia; 2 Univerzitetni Klinicni Center Ljubljana Nevroloska Klinika, Ljubljana, Slovenia

**Keywords:** cognitive assessment, neuropsychiatry, post-stroke cognitive impairment (PSCI), post-stroke depression, stroke rehabilitation

## 1 Introduction

We read with interest the prospective cohort study by Yan et al. (1), which followed 78 acute ischemic stroke survivors for 6 months and examined a composite outcome of post-stroke depression (PSD) and/or post-stroke cognitive impairment (PSCI) as an index of neuropsychological burden after stroke. The authors report that right-hemisphere lesions, higher baseline PHQ-9 scores, and lower baseline MMSE scores independently predicted this outcome. We welcome the integrated view of mood and cognition after stroke, but wish to comment on how the operationalization of PSD and PSCI using brief screening scales influences both risk stratification and the clinical interpretation of the findings. This integrated clinical perspective is also biologically plausible, as the overlap between PSD and PSCI may reflect disruption of prefrontal–subcortical networks, altered monoaminergic signaling, and stroke-related neuroinflammatory processes affecting both mood regulation and cognitive functioning.

## 2 The combined PSD/PSCI endpoint

Combining PSD and PSCI into a single endpoint is clinically attractive, given their frequent co-occurrence and overlapping neurobiological substrates. However, it also merges at least three partly distinct trajectories: predominantly depressive symptoms, primarily cognitive impairment, and comorbid PSD and PSCI. These groups may differ in determinants, prognosis, and treatability. Yan et al.'s subgroup analyses suggest differences between PSD-only, PSCI-only, and comorbid cases in vascular subtype, exercise patterns, and neuropsychological scores. More explicit discussion of this heterogeneity, and exploratory models for PSD and PSCI separately where feasible, would help clinicians judge whether early depressive symptoms mainly predict later depression, cognitive decline, or a broader vulnerability profile.

## 3 Depression screening in the acute stroke setting

The diagnosis of PSD was based on DSM-5 criteria, with PHQ-9 ≥5 used as a screening threshold before clinical interview. This is pragmatic in neurology services, and the subsequent DSM-5 clinical interview confirmation is an important methodological strength, as it mitigates the risk that a low screening threshold alone could misclassify transient post-stroke symptoms as PSD. Nevertheless, such a low PHQ-9 threshold captures very mild symptom levels and may include transient distress, adjustment reactions, fatigue, sleep disturbance, and reduced energy, all of which are common and multifactorial after acute stroke (2–5). At the same time, depressive symptoms identified during the first post-stroke week should not necessarily be regarded as clinically inconsequential merely because they are subsyndromal or transient. Their predictive value for subsequent depressive episodes is itself an important clinical question that longitudinal studies such as that of Yan et al. (1) may help to clarify. Future studies and clinical pathways could also evaluate a two-stage approach, with initial PHQ-2 screening followed by PHQ-9 assessment in screen-positive patients and structured diagnostic interview where clinically indicated, for example at a PHQ-9 score of ≥10.

## 4 Cognitive assessment with MMSE and MoCA

The definition of PSCI was aligned with NINDS-CSN criteria, with impairment in at least one cognitive domain assessed using MMSE and MoCA. While MMSE is familiar and easy to administer, it has recognized limitations in the post-stroke setting, particularly for executive function, processing speed, neglect, and visuospatial deficits that are highly relevant after right-hemisphere lesions (6–8). The MoCA partly addresses these limitations by including tasks of visuospatial/executive function, attention, and delayed recall and may be more sensitive than the MMSE to vascular cognitive deficits after acute stroke. However, as a global screening instrument, the MoCA cannot fully characterize domain-specific cognitive profiles or reliably distinguish focal stroke-related deficits from more diffuse cognitive impairment. For a more precise characterization of post-stroke cognitive profiles, future studies could complement global screening with brief domain-specific measures. Executive dysfunction may be assessed with the Trail Making Test Part B or the Frontal Assessment Battery, while the Wisconsin Card Sorting Test may be considered where time and clinical capacity permit. Processing speed may be assessed using the Symbol Digit Modalities Test or Digit Symbol Coding. Visuospatial impairment and hemispatial neglect could be evaluated using the Rey–Osterrieth Complex Figure, line-bisection tasks, or the Behavioral Inattention Test. The selection and interpretation of these measures should account for aphasia, visual and motor deficits, fatigue, stroke severity, and the practical constraints of acute stroke services. In this cohort, lower baseline MMSE scores are interpreted as indicating pre-existing cognitive vulnerability or reduced reserve. Given the timing of assessment within the first week, and the exclusion of patients with severe premorbid impairment or inability to cooperate, baseline MMSE more likely reflects a mixture of premorbid reserve, acute neurological effects, and emerging PSCI. Framing MMSE as a composite clinical indicator, rather than a pure measure of reserve, would better match neuropsychiatric practice.

## 5 Clinical implications

Despite these caveats, the study supports the early integration of structured mood and cognitive assessment into post-stroke pathways. From a neuropsychiatric perspective, its most important translational message is not only that acute PHQ-9 and MMSE scores may help flag risk, but also that these signals should trigger timely, multidisciplinary follow-up. Future studies could strengthen this message by specifying how neuropsychiatric and psychological expertise might be embedded within stroke units, rehabilitation teams, and follow-up clinics.

## 6 Conclusions and future directions

In summary, Yan et al. contribute to the growing recognition that emotional distress and cognitive vulnerability are intertwined after stroke. Future work could build on these findings by refining the operational definitions of PSD and PSCI, including domain-specific measures of executive function, processing speed, visuospatial function, and neglect, modeling depressive and cognitive trajectories separately where possible, and treating PHQ-9 and MMSE scores as dynamic clinical indicators rather than fixed biomarkers. Repeated assessment during the acute phase and at follow-up may be more informative than a single score, particularly when interpreted as individual trajectories rather than isolated threshold crossings. Worsening, persistently elevated, or newly emerging depressive symptoms should prompt diagnostic reassessment and appropriate psychological or neuropsychiatric follow-up; similarly, persistent or declining cognitive performance should trigger more detailed cognitive and functional assessment, including neuropsychological evaluation where indicated. In contrast, improving scores may support continued monitoring while recognizing that recovery trajectories should be interpreted in the context of stroke severity, disability, aphasia, and other evolving clinical factors. Such refinements may sharpen prognostic tools while keeping them closely aligned with neuropsychiatric diagnostic standards and the lived clinical realities of stroke survivors.
